# Long-term partial response in a patient with liver metastasis of primary adrenocortical carcinoma with adjuvant mitotane plus transcatheter arterial chemoembolization and microwave ablation: a case report

**DOI:** 10.3389/fonc.2023.1157740

**Published:** 2023-05-29

**Authors:** Jianhua Deng, Lihui Wei, Qihuang Fan, Zoey Wu, Zhigang Ji

**Affiliations:** ^1^ Department of Urology, Peking Union Medical College Hospital, Beijing, China; ^2^ Department of Medicine, Genetron Health (Beijing) Co. Ltd., Beijing, China

**Keywords:** adrenocortical carcinoma, liver metastases, mitotane, TACE, WMA, partial response

## Abstract

Adrenocortical carcinoma (ACC) is a rare, heterogeneous, and aggressive malignancy with a generally poor prognosis. Surgical resection is the optimal treatment plan. After surgery, both mitotane treatment or the etoposide-doxorubicin-cisplatin (EDP) protocol plus mitotane chemotherapy have a certain effect, but there is still an extremely high possibility of recurrence and metastasis. The liver is one of the most common metastatic targets. Therefore, techniques such as transcatheter arterial chemoembolization (TACE) and microwave ablation (MWA) for liver tumors can be attempted in a specific group of patients. We present the case of a 44-year-old female patient with primary ACC, who was diagnosed with liver metastasis 6 years after resection. During mitotane treatment, we performed four courses of TACE and two MWA procedures in accordance with her clinical condition. The patient has maintained the partial response status and has currently returned to normal life to date. This case illustrates the value of the practical application of mitotane plus TACE and MWA treatment.

## Introduction

Adrenocortical carcinoma (ACC) is a rare, heterogeneous, and aggressive malignancy with a generally poor prognosis. At least 50% of patients are detected with metastatic tumors at initial diagnosis. The prognosis for ACC is poor, with a 5-year overall survival (OS) rate of 30% (1), depending on the stage of the disease at diagnosis—5-year survival is 60% to 80% for tumors confined to the adrenal space, 35% to 50% for locally advanced disease, and much lower for metastatic disease with percentages reported ranging from 0% to 28% (2). Although ACC is potentially curable in the early stages, only approximately 30% of malignancies are located in the adrenal gland when diagnosed (3, 4). Most tumors have distant metastases, and this aggressive behavior leads to a poor prognosis. The medical treatment options for ACC are limited and mitotane is the only drug available as its good efficacy on prolongation of RFS (HR = 0.62; 95%CI, 0.42-0.94; P < 0.05) and OS (HR = 0.69; 95%CI, 0.55-0.88, P < 0.05) in patients with ACC after radical surgery (5). For advanced or recurrent patients with poor prognostic parameters, a more aggressive treatment regimen (mitotane with chemotherapy) is recommended according to the result of FIRM-ACT, the only randomized controlled trial designed to evaluate the efficacy of mitotane combination with chemotherapy for ACC (6): patients in the etoposide-doxorubicin-cisplatin-mitotane (EDP-M) group had a significantly higher response rate than those in the streptozocin-mitotane group (23.2% vs. 9.2%, P<0.001) and longer median progression-free survival (PFS, 5.0 months vs. 2.1 months, P<0.001). But EDP-M is more toxic (6) and mitotane has a very poor aqueous solubility (7), there is still limited treatment for advanced or recurrent ACC patients and more exploration is needed.

Combination therapy of transcatheter arterial/transarterial chemoembolization (TACE) and microwave ablation (MWA) is a minimally invasive technique performed by interventional radiologists that delivers embolization chemotherapy, injected through a catheter, into the hepatic artery that directly supplies the tumor. Although TACE+WMA is used frequently to treat hepatocellular carcinoma, intrahepatic cholangiocarcinoma, and intrahepatic colorectal cancer metastases, to our knowledge, it is rarely used for the treatment of metastatic ACC (8–11).

We present a case of a 44-year-old female who was diagnosed with stage III ACC. She underwent a radical adrenalectomy of the left adrenal tumor and then received mono-mitotane chemotherapy. Five years after surgery, she was diagnosed with liver metastasis from ACC. During mitotane treatment, she continued to have persistent liver disease progression and, therefore, underwent four courses of TACE+WMA therapy. Following this treatment, the patient experienced a partial response (PR) to treatment and has remained progression-free for more than 28 months until the last follow-up. We present the following case in accordance with the CARE reporting checklist.

## Case description

In 2013, a 36-year-old female Chinese patient presented to the Peking Union Medical College Hospital complaining of infertility, menelipsis for about 4 months, weight gain and facial vellus hair for more than 4 years. She was treated with Yasmin at the local hospital for symptoms of facial vellus hair and elevated testosterone in 2009, then with progesterone due to menopause and reached regular menstruation from July 2011 to 2013 July. In September 2013, she came to Peking Union Medical College Hospital for diagnosis when she developed symptom of menopause again.

**Figure 1:**
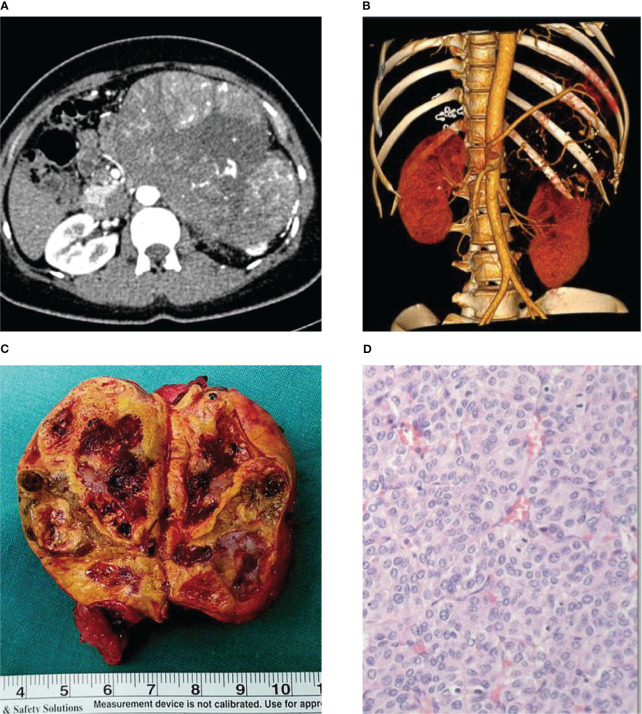
Characteristics of primary adrenal cortex tumor. **(A)** CT scan result of the patient revealed left adrenal gland huge mass. **(B)** CT-3D reconstruction of patient’s abdomen. **(C)** Surgical removal of adrenal cortex tumor. **(D)** Immunohistochemical results of tumor tissue.

Physical examination showed no obvious abnormality, and the patient also denied of family history. Computed tomography (CT) scan and ultrasound examination were preformed that day, which revealed a large mass in the left adrenal gland (**Figure 1A**). And 3D reconstruction of CT scans showed a significant soft tissue burden in the left adrenal area with the size of approximately 15.9 cm × 15.5 cm, the lesion showed a clear boundary and was supplied by the left adrenal artery and the branches of the left renal artery (**Figure 1B**). Blood tests revealed plasma ACTH, TSTO, 24-hour UFC and serum total cortisol levels of 42.7 pg/ml, 171.4 ng/dl, 1094 μg/dl, and 16.29 μg/dl, respectively. It is worth mentioning that DHEA value reached an extraordinarily high level, 6779.0 μg/dl. A fine needle aspiration biopsy (FNAB) sample was also taken, andthe pathology showed Ki67 was 3%, excluded pheochromocytoma. Given all these above, her diagnosis was made as “left adrenal gland occupied, with the possibility of ACC” at that time, and was hospitalized for detailed medical treatment.

**Figure 2:**
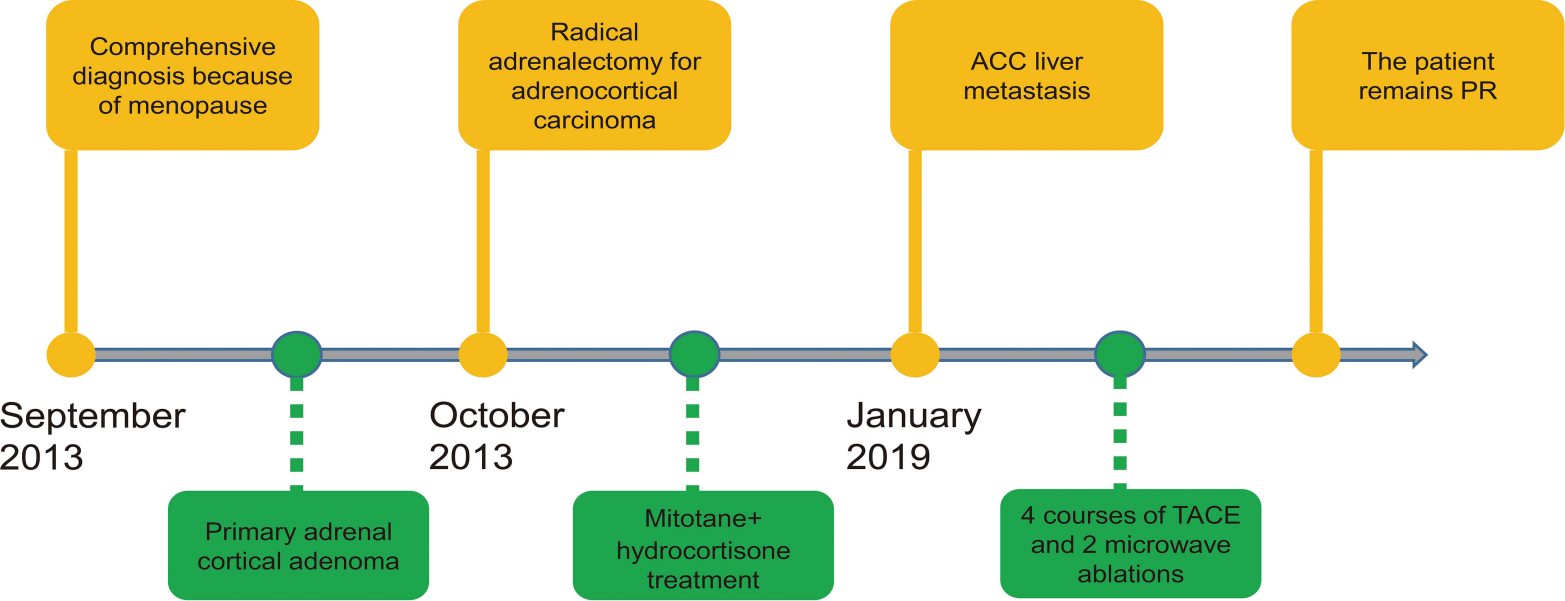
Timeline of this case.

The timeline of the case including the most important therapeutic procedures is shown in **Figure 2**. In October 2013, the patient received radical adrenalectomy for ACC. The postoperative pathology examination of the tumor tissue revealed an adrenal cortex adenoma, which invaded the periadrenal tissue, with large leomorphic cells showing high mitotic rate, atypical mitoses, extensive necrosis, and hemorrhage which was consistent with the pathology of FNAB. The tumor grew actively (the malignant potential could not be determined), and the size was 18 cm × 12.5 cm × 10 cm (**Figure 1C**), determined as stageIII according to the European Network for the Study of Adrenal Tumours (ENSAT) staging system. Tumor immunohistochemistry parameters were CgA (-), Melan-A (+), Syntwo (+), AE1/AE3 (+), P53 (-) (**Figure 1D**), and Ki-67 approximately 3%. The postoperative blood test results were as follows: plasma ACTH, TSTO, 24-hour UFC levels were 30.2 pg/ml, 15.0 ng/dl, and 36.55 μg/dl, respectively, returned to normal levels. Her menstruation returned to normal 1 month after the operation and she underwent routine follow-up at the local hospital.

In January 2019, the patient was evaluated again due to menopause. The color Doppler ultrasound results from the local hospital showed a moderately echogenic mass in the liver. The patient returned to Peking Union Medical College Hospital for further examination in March 2019. Multiple lesions in the liver were observed through abnormal enhanced CT and multiple low-density nodules and tumor shadows. Liver and kidney function examination revealed that K^+^ 5.2 mmol/L, GGT 329 U/L, Alb 45 g/L, ALP 194 U/L, AST 50 U/L, ALT 9 U/L; AFP 2.6 ng/ml. The results described above raised the possibility of ACC liver metastasis. She was treated with hydrocortisone (10 mg q8h) and oral mitotane (1.0 g q8h, plasma concentration 14.1 mg/l) from March 2019. The patient developed diarrhea, sweating, lethargy, and occasional chest tightness after taking mitotane, which was considered as common adverse reactions. To prevent liver damage from mitotane, ESSENTIALE 456 mg tid was used at the same time. However, her condition did not improve significantly and her liver lesions was still enlarged. On the basis of the Clinical Practice guidelines of the European Endocrine Society for the treatment of adult ACC, patients with advanced ACC with metastasis may benefit from local treatment (12), we treated her with TACE followed by MWA for the liver lesions.

**Figure 3:**
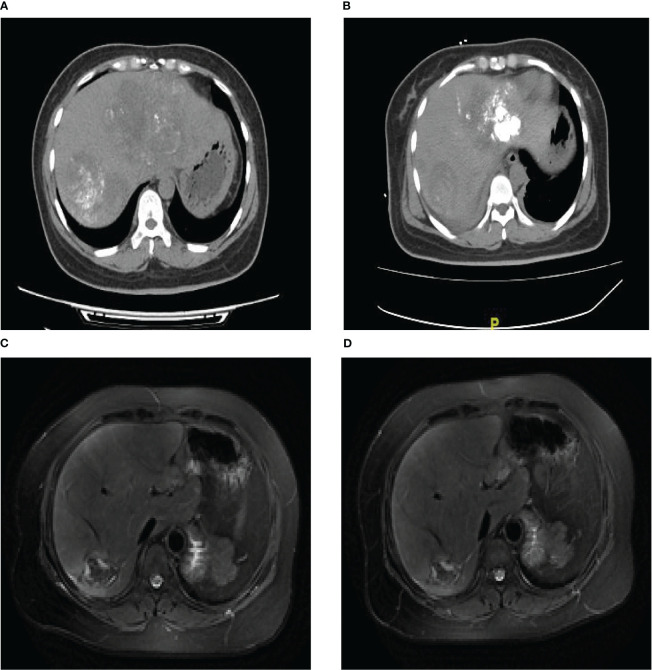
TACE perioperative imaging results. **(A)** CT scan picture of patient after the first TACE. **(B)** CT scan picture of patient after four TACE surgeries. **(C)** The abdomen conventional dynamic enhanced MRI image in March 2022. **(D)** The abdomen conventional dynamic enhanced MRI image in September 2022.

On 4 March 2019, for the first treatment of TACE, a microcatheter was used to infuse a mixture of 10 ml of lipiodol and 5 ml of leroxatin through the superior mesenteric artery to the blood supply artery of the liver tumor and a good deposition effect was obtained (**Figure 3A**). The patient did not experience any side effects and continued to receive mitotane and hydrocortisone treatment. In the next 2 months, the patient completed another three courses of TACE on 3 April, 18 April and 9 May, respectively. All the treatment went smoothly, and the mitotane and hydrocortisone treatment was continued during this period (**Figure 3B**). On 30 May 2019, the patient underwent a CT-guided microwave ablation operation of the liver tumor. Due to pleural hemorrhage in the surgery, peripheral intravenous fluids and a blood transfusion were performed, and pleural effusion drainage and microcatheter super-selected right inferior phrenic artery and right hepatic artery embolization were performed again. On 1 August 2019, the patient completed the second microwave ablation procedure for the liver, and the curative effect was evaluated as partial response(PR) according to RECIST1.1. Subsequently, the patient received continuous mitotane (0.5 g tid) and hydrocortisone treatment. There were no serious adverse reactions for her.

Follow-up examinations were routinely performed after treatment. In the enhanced magnetic resonance imaging (MRI) examination in March and September 2022, her liver showed multiple mass-like abnormal signals, and the lesions were slightly smaller (**Figures 3C, D**). During this period, the patient’s treatment was mitotane (0.5g tid) and hydrocortisone. The evaluation of her treatment outcome as TACE+MWA plus mitotane regimen remained PR, and her condition was also stable and comfortable for about 28 months so far.

## Discussion and conclusion

Although our understanding of treatments has improved in recent years, patients diagnosed with ACC-metastatic disease after surgery continue to experience poor outcomes, with poor overall survival (13). The liver is one of the most common sites of metastases in ACC and contributes significantly to the mortality and morbidity of patients. The management of ACC requires consideration of oncologic and endocrine simultaneously as the special function of the adrenal gland. But it is regrettable that the level of treatment evidence is grade II to grade IV for ACC up to now, which were based on 1 trial, retrospective studies and nonrandomized trials (14). Treatments for ACC are still being explored and there is limited evidence regarding liver-directed therapy in the treatment of patients with metastatic ACC. Studies have shown that systemic antitumor therapy combined with local therapy is expected to achieve higher tumor response (15).

Mitotane is a commonly used agent preferably for patients with unresectable or recurrent ACC due to its high object response rate. A study evaluated the therapeutic effect of mitotane in the treatment of 391 advanced ACC patients, and found 26 (20.5%) patients had objective response, including 3 complete responses. The overall median PFS and OS was 4.1 and 18.5 months, respectively, demonstrating that mitotane is effective against advanced ACC (16). In recent years, local treatment has been advocated for the treatment of metastatic ACC. Liver-directed therapies have already been proven to improve treatment response in other tumors affecting the liver, such as hepatocellular carcinoma and colorectal tumors (17, 18). There are also some ACC patients with liver metastases benefited from local therapy. A study was reported to treate 29 patients with ACC with liver metastases by TACE. The median PFS was 9 months, and the median OS was 11 months after the first procedure (19). Another study published in 2009 described two patients with liver metastases after being diagnosed with ACC who underwent transcatheter arterial embolization (TAE) for liver lesion. Both patients achieved complete responses after liver-directed therapy (20) and the OS was 77 and 51 months, respectively. The characteristics and outcomes of 3 patients were listed in **Table 1**. The treatment for liver lesion was different in Hideo’s report and ours: we treated liver lesion with TACE with followed by MWA, which was reported could improve local control (21), prolong OS (22) and has better clinical effectiveness (23) compared with TACE monotherapy in liver cancer. And mitotane was used for systemic antitumor therapy concomitantly. Beyond that, the site, age, interval until recurrence or metastasis and DHEA are all different between these patients, which may also affect the curative effect in some degree, and more cases are needed to summarize the clinical treatment experience for ACC with liver metastasis.

**Table 1:** Characteristics and Outcomes of 3 patients with ACC.

	1^*^	2^*^	3^#^
Site	right	right	left
Age	57	54	36
Gender	Female	Male	Female
Symptoms	–	back pain	menopause, weight gain and facial vellus hair
Manifestation of tumor growth	–	+	+
Hypertension	+	–	–
Hormonal activity	Cushing’s syndrome	–	–
Size of tumor, cm	7	20	15.9
Ki-67	–	–	3%
DHEA, μg/dl	–	–	6779
Tumor weight, g	32	3080	2150
pT	2	3	3
Postoperative stage	II	III	III
Adjuvant therapy	–	–	–
Interval until recurrence or metastasis, months	33	6	62
Location of recurrence or metastasis	liver (solitary)	liver (solitary)	liver (solitary)
Treatment for recurrence or metastasis	mitotane+cytotoxic chemotherapy→TAE	mitotane+cytotoxic chemotherapy→TAE	mitotane+TACE+MWA
Effect	CR	CR	PR
Survival period, months	77	51	108(still alive as of press time)
Prognosis	AWM	NED	AWM

1.*patients from Hideo’s report[];#patient of our case.

2.NED, no evidence of disease; DOD, death of disease; AWM, alive with metastasis; CR, Complete response; PD, progressive disease; PR, partial respons.

3.–, absence; +, presence.

Here we reported a case that advanced ACC with liver metastases treated with systemic antitumor therapy combined with local therapy for liver lesion (motatine combined with TACE+MWA). The treatment outcome was PR and the PFS of the patient has been extended to about 28 months so far she was still alive up to now (September 2022). Based on our review of the limited literature available, liver-directed therapies such as TACE or SIRT have significant potential as part of treatment regimens in patients with metastatic liver burden in ACC. We consider this to be an emerging area and further research is needed to guide treatment decisions. With systemic antitumor therapy combined with local therapy, such as mitotane plus TACE+MWA we used in this case with less toxicity and side effects in patients with high-grade disease, traditional ACC treatment decisions may achieve better results, which we used in this case. We suggest that there is a need for further research including clinical trials on this topic to further clarify the role of TACE in these patients.

## Data availability statement

The original contributions presented in the study are included in the article/supplementary material. Further inquiries can be directed to the corresponding author.

## Ethics statement

Written informed consent was obtained from the individual(s) for the publication of any potentially identifiable images or data included in this article.

## Author contributions

ZJ designed and supervised the study. JD, LW and QF searched the literatures and participated in clinical data acquisition. JD and LW drafted the manuscript. JD, QF and ZW gave critical revision of the manuscript for important intellectual content. All authors contributed to the article and approved the submitted version.
